# Pathologic Conditions of the Pharynx and Contiguous Structures during Early Childhood, Prime Factors in the Etiology of Malformed Maxillæ and Irregular Teeth, Etc.

**Published:** 1898-05

**Authors:** William A. Mills

**Affiliations:** Baltimore, Md.


THE
AMERICAN JOURNAL
—OF—
DENTAL SCIENCE.

VOL. XXXII. BALTIMORE, MAY, 1898. No. 1.

### Selections.

ARTICLE I.

### PATHOLOGIC CONDITIONS OF THE PHARYNX AND CONTIGUOUS STRUCTURES DURING EARLY CHILDHOOD, PRIME FACTORS IN THE ETIOLOGY OF MALFORMED MAXILLÆ AND IRREGULAR TEETH, ETC.

WILLIAM A. MILLS, D. D. S., BALTIMORE, MD.

Read before the Section on Stomatology, at the Forty-eighth Annual Meeting of the American Medical Association, held at Philadelphia, Pa., June 1-4, 1897.

The physiologic functions of the nasal cavities are as follows: "They elevate the temperature of the inhaled air, give it moisture, and purify it by arresting all particles of dust and other substances which it may contain;" they act as resonance cavities for the voice and are also the seat of the sense of smell.

A professional experience of more than twenty-five years, has taught me that any imflammatory lesions found in children between the ages of 4 and 12 years which obstruct these natural conditions, are in the majority of cases, the chief agents in the causation of malformed jaws, and the consequent abnormal alignment of the teeth. (To the same cause may be attributed beaked or hooked noses, so often found in connection with these deformities.) I have reached this conclusion after many years of ceaseless endeavor to trace out cause and effect. Others may theorize differently, but theory alone will not stand, unless proven clinically correct.

I cite the following case in my practice:

A male child over 7 years old was brought to have a tooth filled, which was supposed to cause pain in the right ear and the right angle of the inferior maxilla.

The patient presented the following clinical conditions: Almost complete obstruction of the nasal cavities; face pale, anemic and haggard; features contracted at the angles of the jaws and beneath the malar processes, giving the characteristics of the mouth-breather, or "dog-face," as Dr. Cathell terms them. I found a small cavity in a molar tooth, which in no way could have caused the pain. This was filled but gave no relief. I depressed the tongue and examined the tonsils. These I found so greatly enlarged that they almost met at the median line. I was informed that deglutition was both difficult and painful. When the tongue was forced well down with a tongue depressor, quite a large quantity of yellow pus sprang from an unseen source in the right tonsil. On the removal of the depressor, the patient exclaimed. My pain has gone!

On examining the superior maxilla, I noticed a very perceptible contraction on both sides, anterior to the first permanent molar teeth, with a decided inclination of the roof of the mouth to become elevated.

I informed the aunt of the state of affairs, and that unless heroic measures were quickly taken for the child's relief, by a rhinologist, very serious consequences would be sure to follow.

Six months later the patient returned, and I was informed that notice had been taken of previous instructions and a specialist consulted the same day. He had removed some adenoid growths, and was still reducing the tonsils with the galvano-cautery. He was now a bright and rosy-faced boy, with normal respiration, and we found no sign of the former contraction of the jaws, or rising of the arch, all of their outlines being perfectly normal.

This particular case demonstrated that either the adenoid vegetations, or the enlarged and diseased tonsils, or both, were the prime factors in the causation, not only of the malformations of the maxillæ, but also the oral respiration.

If a correct diagnosis had not been made, and only palliative treatment been given, there is no doubt that a case of V, or saddle-shaped arch would have been developed, with irregular teeth as concomitants; especially as the patient's parents and grandparents are full-blooded Germans, and have large jaws and teeth.

I have often found similar conditions in young patients, whose parents thought so little of the matter that they deemed no treatment necessary, as the children never complained of pain; while others "made their own diagnosis, and treated for conditions which did not exist, being ignorant of all possible results." Thus, from time to time, the lesions were permitted to progress, until more acute symptoms developed, when the family physician was called; and as is often the case in such diseases, he failed to make a correct diagnosis, or give the proper treatment, and thinking the disturbance of no great importance only prescribed some simple wash or nasal douche, thus allowing inflammatory conditions, which could have been cured, to develop into more chronic forms with all their evil consequences.

The starting point in the majority of these cases, has its origin in some slight irritation of the mucous membranes of the throat, especially the tonsils. "It seems remarkable," says an authority, "that the fauces at this time of life, should be the seat of so many inflammatory processes."

To show with what indifference some practitioners treat such cases, I cite the following: A reputable physician, whose daughter, aged 14 years, has nasal catarrh, V-shaped arch, irregular teeth and an almost complete obstruction of the nasal cavities, when told that he should do something for her relief replied, "She will come around all right when she gets older;" and this seems to be the opinion with many general practitioners.

In conclusion, I would urge upon both medical, and dental practitioners the importance of being ever on the alert to discover the first signs of any inflammatory manifestation in the throat or nasal cavities of children; as so many inflammatory disturbances are prone to develop in the respiratory passages, without in themselves showing any very acute symptoms, and if permitted to continue without proper treatment, cause disease and malformations which in some cases defy all remedial agencies.

When a patient has a slight occasional cough or clearing of the throat, any peculiarity of intonation, huskiness of voice or nasal twang, an examination should be made to discover the cause, and when located, treatment given at once; and if oral respiration is fully established, some reputable specialist should be recommended, as he is better qualified to make a correct differential diagnosis, and prescribe the best remedies. I believe that if this is done, fewer cases of malformed maxillæ and irregular teeth will be found in the future than at present.

The following are a few replies to personal letters sent, asking for opinions as to the etiology of malformed maxillæ and irregular teeth.

Dr. John Nolan Mackensie, Johns Hopkins Hospital, says: "Rhinologists generally hold that the deformities in question are sometimes produced by nasal and post-nasal obstructions (deflection of the septum and adenoid growths being two conspicuous examples). Indeed it has been shown experimentally that asymmetric conditions of the corresponding side of the cranium may be produced in young guinea-pigs by artificially occluding the nostril. Malnutrition must of course be at the bottom of the matter, due probably to defective blood supply, both in quantity and quality."

Dr. M. H. Cryer says: "It is my opinion, that an inflammation of the tonsil and surrounding tissue will cause tension of the palato-pharyngeal and palato-glossus muscles; if this be so, they would naturally pull the lateral portions of the arch downward and inward; especially is this the case with children when their bones are soft and yielding."

Dr. Harrison Allen says: "Probably the most important factor present is oral respiration. Disuse of important functions (instanced here by the nasal passages) invariably excite disturbance. No 'starvation' of the tissue is present in these cases as I understand them, nor is there disease, as the phrase is usually employed. I do not think undue pressure of the muscles against the jaws exists, nor are the jaws themselves weakened, except that they may be weakened by having their curves of normal action interfered with."—*Journal Am. Med. Asso.*

302 Dolphin Street.

